# Improvements in Reconstructive Surgery of the Head

**Published:** 1919

**Authors:** 


***Improvements in Reconstructive Surgery of the Head.*** By JOHN B. ROBERTS. *Surgery, Gynecology and Obstetrics*, October, 1918.

Surgeons are gradually coming to appreciate the immense value of prompt plastic reconstruction.

Practically all tissues can be repaired by plastic procedures, if the operative technic is mechanically efficient, provided that the wounds be kept aseptic from the moment of their production to the end of the period demanded by nature for the metabolic reestablishment of function.

The greatest adversary in plastic surgery is bacterial infection. Tension on sutures and too early disturbance of the repaired tissues should be avoided as carefully as possible.

The usual methods are :

A. Displacement : (1) approximation ; (2) sliding to transfer tension.

B. Interpolation, borrowing material from vicinity, from another region, or from another person or animal.

C. Enthesis : burying in the tissues foreign bodies, such as paraffin, wire, metal, glass, rubber.

D. Retrenchment : cutting out ellipses or wedges.

E. Substitution.

F. Strategic : temporary displacement, as mandible to get at pharynx ; zygoma and cranium to reach brain.

In complicated procedures, a series of operations extending over many months may be necessary ; too much should not be attempted at a time.

*New Ideas in Plastic Surgery of the Head.* Prompt readjustment of tissues and immobilization are two important factors in war surgery.

Mandibular war fractures are usually both comminuted and infected. Immediate removal of all foreign bodies, displaced teeth, unattached splinters of bone, and prompt fixation are most advisable.

If there is a considerable loss of bone in the lower jaw, the free ends should be kept apart with some temporary spreading dental apparatus to prevent dragging of them together by means of muscular contraction. Then after a few weeks a permanent prosthetic appliance may be adapted.

Control of a detached tongue may be obtained with a suture through its base, knotted around the hyoid bone.

Ptosis of the upper eyelid may be relieved with success by a muscle substitution operation when the fibers of the occipitofrontalis have their normal innervation. A band of muscle from the frontalis is turned down and passed through a tunnel beneath the skin of the brow so as to be fixed with sutures to the upper edge of the tarsal cartilage.

The author emphasizes the high value of Masland osteoplastic electric saw. It is guided with one hand with almost the convenience of a pocket knife.

The author devised, 30 years ago, an electrically driven burr or trephine, most convenient whenever a preliminary hole of entrance is required.

